# Frontiers of torenia research: innovative ornamental traits and study of ecological interaction networks through genetic engineering

**DOI:** 10.1186/1746-4811-9-23

**Published:** 2013-06-26

**Authors:** Masahiro Nishihara, Takeshi Shimoda, Takashi Nakatsuka, Gen-ichiro Arimura

**Affiliations:** 1 Iwate Biotechnology Research Center, Kitakami, Iwate 024-0003, Japan; 2 National Agricultural Research Center, Tsukuba, Ibaraki 305-8666, Japan; 3 Department of Biological and Environmental Science, Graduate School of Agriculture, Shizuoka University, Shizuoka 422-8529, Japan; 4 Department of Biological Science & Technology, Faculty of Industrial Science & Technology, Tokyo University of Science, 6-3-1 Niijuku, Katsushika-ku, Tokyo 125-8585, Japan

**Keywords:** Flavonoid, Flower color, Herbivore-induced plant volatiles (HIPVs), Indirect defense, Metabolic engineering, Torenia

## Abstract

Advances in research in the past few years on the ornamental plant torenia (*Torenia* spps.) have made it notable as a model plant on the frontier of genetic engineering aimed at studying ornamental characteristics and pest control in horticultural ecosystems. The remarkable advantage of torenia over other ornamental plant species is the availability of an easy and high-efficiency transformation system for it. Unfortunately, most of the current torenia research is still not very widespread, because this species has not become prominent as an alternative to other successful model plants such as Arabidopsis, snapdragon and petunia. However, nowadays, a more global view using not only a few selected models but also several additional species are required for creating innovative ornamental traits and studying horticultural ecosystems. We therefore introduce and discuss recent research on torenia, the family Scrophulariaceae, for secondary metabolite bioengineering, in which global insights into horticulture, agriculture and ecology have been advanced. Floral traits, in torenia particularly floral color, have been extensively studied by manipulating the flavonoid biosynthetic pathways in flower organs. Plant aroma, including volatile terpenoids, has also been genetically modulated in order to understand the complicated nature of multi-trophic interactions that affect the behavior of predators and pollinators in the ecosystem. Torenia would accordingly be of great use for investigating both the variation in ornamental plants and the infochemical-mediated interactions with arthropods.

## Introduction

Torenia spps. are dicotyledonous plants that belong to the class Magnoliopsida, order Scrophulariales and family Scrophulariaceae, and include annuals and perennials. Torenia is the common name for several species in the genus *Torenia* (*e.g. T. fournieri* Lind., *T. concolor* Lind., *T. asiatica* L. and *T. hybrida* [*T. fournieri* x *T. concolor*]) [1]. In addition, *T. fournieri* and its hybrids, such as *T. hybrida*, are often called wishbone flowers or blue wings. Almost all species of torenia occur in tropical and subtropical Asia, Africa or Madagascar [2]. There are 50 species, 20 of which are from Cambodia, Laos and Vietnam, and 19 from Thailand [2]. Many hybrids have been produced in the last 30 years, with a variety of flower colors ranging from white with yellow throats to blue, cobalt, lavender and violet. However, torenia is not only a horticultural plant but also an experimental one with several useful characteristics. These include ease of genetic transformation, ability to differentiate adventitious shoots and roots, and capacity for *in vitro* flowering. Especially, a high-efficiency *Agrobacterium*-mediated transformation system has been established in torenia, and this gives torenia a great advantage when conducting basic research using transgenic plants. The method can achieve a transformation rate of approximately 5% [3]. The short generation time and small plant size of torenia make it possible to reduce the space and effort needed for maintenance compared with other ornamental model plant species such as the snapdragon and petunia. Torenia plants can be propagated vegetatively by stem cuttings, making it easy to obtain sufficient amounts of samples in a short time. Also useful is its small genome size of 171 Mb [4], similar to that of Arabidopsis [5].

In addition, because torenia is a unique plant with a protruding embryo sac, it has been possible to establish an *in vitro* system for observing the guidance of pollen tubes using the naked embryo sac [6]. This feature has put torenia in the forefront of research on the fertilization process in higher plants in which torenia and Arabidopsis were used together for studies that, for instance, revealed the function of *AtLURE1* peptides as pollen tube attractants [7]. In spite of these advantages, most torenia research is, unfortunately, not widespread over the world but rather remains restricted to a few research groups, mainly in Japan.

Flower color is one of the most noteworthy characteristics of ornamental plants, including torenia. Consequently, much effort has been devoted to understanding the molecular and biochemical mechanisms underlying pigment formation in flowers. Early research using transgenic plants indicated that the main pigments in torenia flowers consist of visible flavonoids, *i.e.*, anthocyanins [8]. Torenia flowers also contain unpigmented or pale yellow flavonoids, flavones. This knowledge has allowed the successful modification of torenia flower color by genetic engineering. Torenia has thus joined the group of ornamental horticultural model plants in which flower color modification has been successfully achieved, such as *Petunia hybrida* (petunia), *Dendranthema grandiflorum* (chrysanthemum), *Dianthus caryophyllus* (carnation) and *Rosa hybrida* (rose) [9]. A particular advantage of using torenia as a model system is that it is related to the snapdragon, *Antirrhinum majus,* which has been a model plant in biochemical and developmental genetics for 80 years [10]. Since torenia is easy to transform and belongs to the same family (Scrophulariaceae) as snapdragon, torenia is an excellent platform for testing the function of genes isolated from torenia itself as well as those isolated from snapdragon.

Floral and foliar odors have been little investigated in torenia compared to the large amount of research on petunia and snapdragon. These two plants have been in the forefront of genetic and biotechnological applications, resource development (mutants, transcriptome datasets, genome and EST information) as well as floral studies [11-14]. We provided novel insight into the effects of foliage volatiles on ecological interactions between torenia plants and arthropods [15]. Indeed, such insights are critical for assessing the impact of metabolic engineering of volatiles on horticultural pest control, because volatiles affect the behavior of herbivores, carnivores, flower-visiting generalist predators and parasitoids (of the herbivores), and pollinators.

### Significance of the flavonoid biosynthetic pathway in torenia flowers

**Figure 1:**
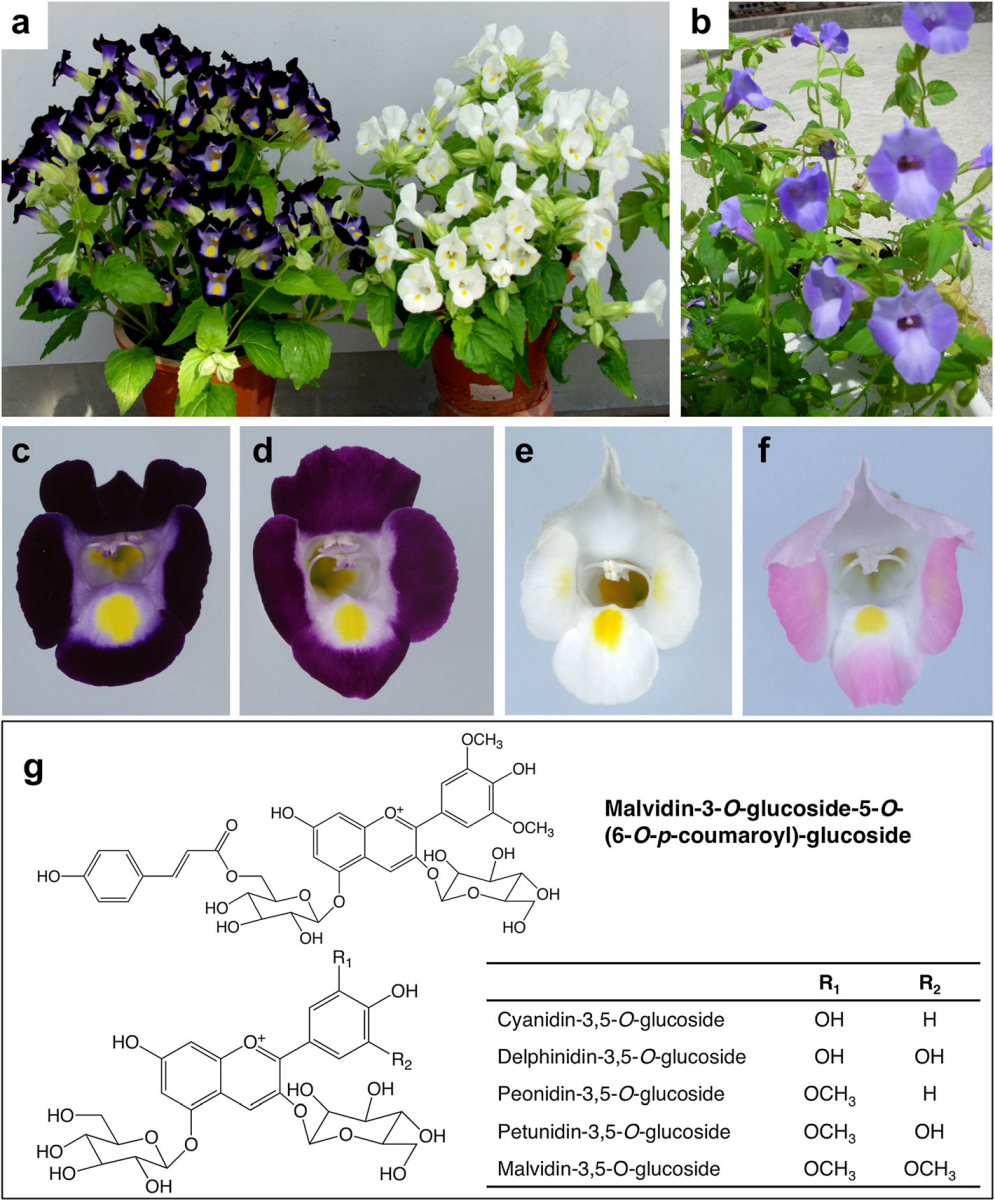
**Genetic engineering of torenia flower color.** Various species of torenia used for transformation: *Torenia fournieri* cv. Crown violet (Left in **a**)), Crown white (Right in **a**)) and *T. hybrida***b**). **c**) Flower of Crown violet. **d**) Transgenic flower of Crown violet with reduced flavone derivatives. **e**) Flower of Crown white. **f**) Transgenic flower of Crown white that accumulated pelargonidin derivatives. **g**) Representation of torenia floral anthocyanins.

Wild torenia species have flowers that range in color from blue to violet, and some of the species such as *T. fournieri* have a yellow splotch in the center. These colors are predominantly due to flavonoid pigments. The flavonoid biosynthesis pathway branches from the phenylpropanoid pathway leading to lignins and phytoalexins. Flavonoids are classified into several subclasses, such as flavones, flavonols, proanthocyanins, and anthocyanins [16]. Of these, anthocyanins and flavones in particular accumulate in torenia petals. For example, the blue petals of *Torenia hybrida* Summerwave blue contain mainly malvidin 3-*O*-β-D-glucoside-5-*O*-(6-*O*-*p*-coumaroyl)-β-D-glucoside together with minor anthocyanins, malvidin 3,5-diglucoside and peonidin derivatives (Figure 1) [17]. The violet petals of *T. fournieri* Crown violet accumulate five anthocyanins, delphinidin 3,5-*O*-diglucoside, cyanidin 3,5-*O*-diglucoside, petunidin 3,5-*O*-diglucoside, peonidin 3,5-*O*-diglucoside and malvidin 3,5-*O*-diglucoside (Figure 1) [8]. This cultivar contains, in addition, three major flavones, luteolin 7-glucoside, luteolin 7-glucuronide and apigenin 7-glucuronide. The flavonoid biosynthetic pathway has been well studied and the genes related to it have also been identified in petunia, snapdragon, Arabidopsis and maize [18]. Flavonoids also play extensive roles in various biological and environmental responses. In particular, they have a crucial role in plant-insect interactions [19].

The phenylpropanoid pathway, leading from phenylalanine to *p*-coumaroyl-CoA, is the entry point to downstream pathways including flavonoid biosynthesis. Structural genes required for flavonoid biosynthesis have been characterized in a variety of plant species, including torenia, as mentioned above. Transcriptional regulation of flavonoid biosynthesis is a particularly active research topic, and notably shows combinatorial regulation by the MYB/bHLH/WDR (MBW) transcriptional complex in a suite of plant species [20,21]. Such a tripartite MBW complex was found to regulate anthocyanin biosynthesis in petunia flowers [22] and grapes [23], and proanthocyanidin (PA) accumulation in Arabidopsis seed coats [24]. Moreover, *ANTHOCYANIN2* (*AN2*), a well-defined MYB-type transcription factor that is a major determinant of flower color variation in petunia, has important effects on pollinator preference. Variation in *AN2* homologues may also account for flower color variation in more distantly related taxa, such as snapdragon [25], suggesting that variation in highly specific transcription factors may be a major source of natural phenotypic variation and perhaps the favored target of natural selection in other species as well [26]. This is in line with the significance of gene duplication of anthocyanin-regulating MYB transcription factors as a genetic basis of the flower color diversification found in the *Mimulus* genus, which contains five species native to central Chile [27]. More recently, it was reported that the genes *TfMYB1* and *TfbHLH1* are expressed in torenia flowers and involved in anthocyanin biosynthesis [28].

The genera of Scrophulariaceae are mainly insect pollinated. For instance, *T. fournieri* is known to be mainly pollinated by stingless bees in Thailand, and potentially by hummingbirds in North America [1]. Each class of pollinator exhibits a particular color preference. Bees typically tend to prefer blue, purple and mauve flowers, whereas butterflies choose pink and red flowers [29]. The flowers of *T. fournieri* exhibit most of these flower colors, and bumblebees, attracted by the floral characteristics, play a role in the pollination [30]. Bee vision is trichromatic but more sensitive to short wave length light than human vision. Bees, therefore, can see not only colors created by anthocyanins visible to humans but also those in the ultra-violet range that humans cannot see. This means that bees can detect nectar guides, which are ultra-violet absorbing zones on petals that attract particular pollinators [31]. Nectar guides contain, in particular, flavonols and flavones which have intense spectral absorption in the 340-380 nanometer region [31]. Flavones are likely to act as copigments, because considerable amounts of flavone derivatives accumulate in torenia petals and a loss of dihydroflavonol 4-reductase (DFR) function in torenia causes flavone accumulation, making the transgenic flowers bluer [8,32]. However, the yellow splotch of torenia flowers is a prominent mark and may function specifically to guide the most effective pollinators to the pollen. Yellow pigmentation is attributed to carotenoids in several torenia cultivars (Crown violet, Lemon drop, and Suzie wong), and carotenogenic genes are responsible for the carotenoid biosynthesis that coordinates petal carotenoid variegation (pers. comm. Dr. Sanae Kishimoto, National Institute of Floricultural Science, Japan).

### Modulation of torenia floral traits by genetic engineering

A simple and efficient transformation system has been established in torenia and therefore various transformation studies have targeted ornamental characteristics in this plant during the past 15 years [5]. These studies have produced useful phenotypic modifications for ornamental traits in flowers such as color, pattern, shape, size, and longevity. In ornamental plants, including torenia, genetic transformations have been applied for producing novel flower colors mainly by metabolic engineering of the flavonoid biosynthetic pathway [33,34].

Application of transgenic techniques to torenia has produced a diversity of flower colors, including white, yellow, pink and red instead of the original violet or blue color in cultivars of *T. fournieri* or *T. hybrida* (Table 1). The genes for flavonoid biosynthesis (*e.g.*, chalcone synthase (*CHS*) and *DFR*) were manipulated to reduce flower pigmentation by down-regulation of these genes with cosuppression or antisense strategies [8,17,35,36] and, more recently, by RNA interference (RNAi) technology [37,38]. Similarly, overexpression and/or downregulation of a suite of genes involved in the flavonoid biosynthetic pathway resulted in variation of flower colors in torenia [32,39,40]. In addition to these classical strategies, a recent innovation has been to divert the metabolic flux towards non-native pigments (e.g., aurone). The coexpression of chalcone 4'-glucosyltransferase (*Am4'CGT*) and aureusidin synthase (*AmAS1*) genes, responsible for the biosynthesis of the yellow aurone pigment in snapdragon, and knockdown of a toreniaflavanone 3-hydroxylase gene (*F3H*) or *DFR* successfully produced aureusidin 6-*O*-glucoside and thus yellow flowers [41].

**Table 1:** Summary of flower color modification of torenia plants by genetic engineering

**Target material**	**Gene and method**	**Gene origin**	**Flower phenotype**	**Reference**
*Torenia fournieri*				
cv. Crown violet	Sense *CHS* or *DFR*, antisense *CHS* or *DFR*	Torenia	White to pale blue	[8,35,36]
cv. Crown reddish-purple	Sense *CHS* or *DFR*, antisense *CHS* or *DFR*	Torenia	Wavy-patterned	[8,35,36]
cv. Common violet	Sense *CHS* or *DFR*, antisense *CHS* or *DFR*	Torenia	Wavy-patterned	[8,35,36]
cv. Crown violet	*GPT*-RNAi	Torenia	Lighter colored	[39]
cv. Crown violet	*DEF*-SRDX	Torenia	Partially decolorized	[50]
cv. Crown violet	Transcription factors-SRDX	Arabidopsis	Various color patterns	[43,44,48]
cv. Crown violet	*MYB24*-SRDX	Arabidopsis	Lacked color at both sides of the petal	[47]
cv. Crown violet	*TCP3*-SRDX	Arabidopsis	Various color patterns	[46]
*T. hybrida* (*T. fournieri* x *T. concolor*)				
cv. Summerwave blue	Sense *CHS*, sense *DFR*	Torenia	White to pale blue	[17]
Inbred line T-33	Sense *CHS* or *DFR*, sense *F3′5′H*	Torenia	Yellow, pink	[17]
cv. Summerwave blue	Sense *F3′H*, sense *F3′5′H*	Torenia	Reddish	[32]
cv. Summerwave blue	Sense *FNSII*	Torenia	Pale blue	[32]
cv. Summerwave blue	*CHS*-RNAi	Torenia	White to pale blue	[37]
cv. Summerwave blue	Sense *AS1* and sense *4′CGT*	Snapdragon	Yellow	[41]
	*F3H*-RNAi, *DFR*-RNAi	Torenia		
cv. Summerwave blue	Sense or antisense *ANS*, *ANS*-RNAi	Torenia	White to pale blue	[38]
cv. Summerwave blue	*F3′H*-RNAi, *F3′5′H*-RNAi	Torenia	Pink	[40]
	Sense *DFR*	Rose or pelargonium		
cv. Summerwave violet	*F3′H*-RNAi, *F3′5′H*-RNAi	Torenia	Darker pink	[40]
	Sense *DFR*	Rose or pelargonium		

Abbreviations: *ANS* anthocyanidin synthase, *AS* aureusidin synthase, *4′CGT* chalcone 4-*O*-glucosyltransferase, *CHS* chalcone synthase, *DEF* DEFICIENS, *DFR* dihydroflavonol 4-reductase, *F3H* flavanone 3-hydroxylase, *F3′H* flavonoid 3′-hydroxylase, *F3′5′H* flavonoid 3′,5′- hydroxylase, *FNSII* flavone synthase II, *GLO* GLOBOSA, *GPT* glucose 6-phosphate/phosphate translocator, *TCP* TEOSINTE BRANCHED1/CYCLOIDEA/PROLIFERATING CELL FACTORS 1/2.

Chimeric REpressor gene-Silencing Technology (CRES-T; [42] has notably been applied to torenia and other floricultural plants such as chrysanthemum, gentian, cyclamen, lisianthus, and morning glory [43,44]. In this system, intriguingly, the transcription factor is able to switch from being an activator to being a repressor by fusion to the EAR motif (SRDX), consisting of only 12 amino acids originally derived from SUPERMAN, which acts as strong repressor [45]. Because chimeric repressors can suppress expression of the target genes even in the face of active endogenous transcription factors, transgenic plants expressing the chimeric repressors produce phenotypes similar to those from loss-of-function mutants in the CRES-T system. In general, efficient suppression of redundantly expressed genes and genes in different plant species is rarely achieved by genetic engineering. However, the CRES-T system, an efficient gene suppression system, offers a powerful tool for this purpose. For example, distinctive color patterns in torenia and *Chrysanthemum morifolium* flowers have been created as a result of reduced anthocyanin accumulation caused by the expression of the repressor of Arabidopsis TCP3 transcription factor [46]. Petals inside the flower buds also exhibited a distinct color pattern in transgenic torenia plants as a consequence of overexpression of the Arabidopsis *MYB24* repressor with a transcriptional repression domain (*MYB24-SRDX*) [47]. Of interest is that transgenic torenia plants with variously modified traits have been produced by the collective transformation of about 50 transcription factors using CRES-T [48]. Moreover, morphological change in the shapes and sizes of flowers or leaves has been achieved by the overexpression of chimeric repressors of the B- and C-class homeotic genes. AGAMOUS (AG) terminates the floral meristem and promotes the development of stamens and carpels in Arabidopsis*,* but transgenic torenia plants expressing AG-SRDX showed pleiotropic changes such as serration in petal margins, anthocyanin accumulation and morphological change in the stigma surface, and formation of extra vascular bundles in petals and styles [49]. Up or down-regulation of these orthologue genes in torenia, *TfGLO* and *TfDEF*, also resulted in various interesting phenotypes such as purple-stained sepals, or serrated petals and partially decolorized petals [50].

Finally, it should be taken into account that, in addition to genetic engineering based on gene delivery, pigment mutations are also produced in torenia by heavy ion beam irradiation [51-53]. Moreover, mutations of petal color are also caused by gamma irradiation [54]. These findings suggest that combining transgenic strategies and radiation breeding would greatly facilitate changes in several flower traits. For instance, the torenia mutant (no. 252), generated by ion-beam irradiation of a transgenic torenia with modified flower color, formed flower buds but did not open flowers, whereas wild-type torenia plants usually open flowers until flower buds have developed at the upper joint [55]. This mutant exhibited a missense mutation in the coding region of the *UFO (UNUSUAL FLORAL ORGANS*) gene that induced a sepaloid phenotype in which the second whorls were changed to sepal-like organs [55].

Insertional mutagenesis provides a powerful tool for studying gene functions by the forward genetic approach, and especially retrotransposons have several advantages over traditional DNA-type insertion elements (Kumar and Hirochika 2001). It is also likely that transposon-tagging in torenia may be possible, as we have recently identified a retrotransposon in genes required for anthocyanin synthesis (Nishihara et al., unpublished). Similar approaches have been used successfully in *Lotus japonicus*[56] and rice [57,58] using the *LORE1* and *Tos17* transposons, respectively. Moreover, a DNA-type transposon (*Ttft1*) that belongs to the En/Spm superfamily has been identified in EMS-induced torenia mutants (Nishijima et al., 2013) and this element might be also useful for transposon tagging in torenia. There has been little thorough investigation, however, of other transient assay systems, such as agro-infiltration, biolistics and virus-induced-gene-silencing (VIGS), that are of use in other plant species. However, novel methods for transient foreign-gene introduction into torenia cells have been developed using ArF excimer laser-induced shock waves [59].

### Modulation of defense properties in torenia by genetic engineering

Plants produce a diversity of volatile compounds from their flowers, leaves and other organs. These compounds include terpenoids, phenylpropanoids/benzenoids, fatty acid derivatives and amino acid derivatives. These compounds play a number of roles in the interaction of plants with the environment [60,61]. Floral volatiles are well known to attract pollinators and can also function as repellants against herbivores or as phytoalexins against plant pathogens [62]. Also of interest is that leaf volatiles emitted from plants damaged by herbivorous arthropods (HIPVs: Herbivore-induce plant volatiles), attract natural enemies of the herbivores [63]. These volatiles therefore function as indirect plant defenses against herbivores. HIPVs also induce or prime defense responses against future herbivore attack in neighboring plants [64,65], influence the behavior of insect herbivores or pollinators [66], and interfere with infection by plant pathogens [66].

**Figure 2:**
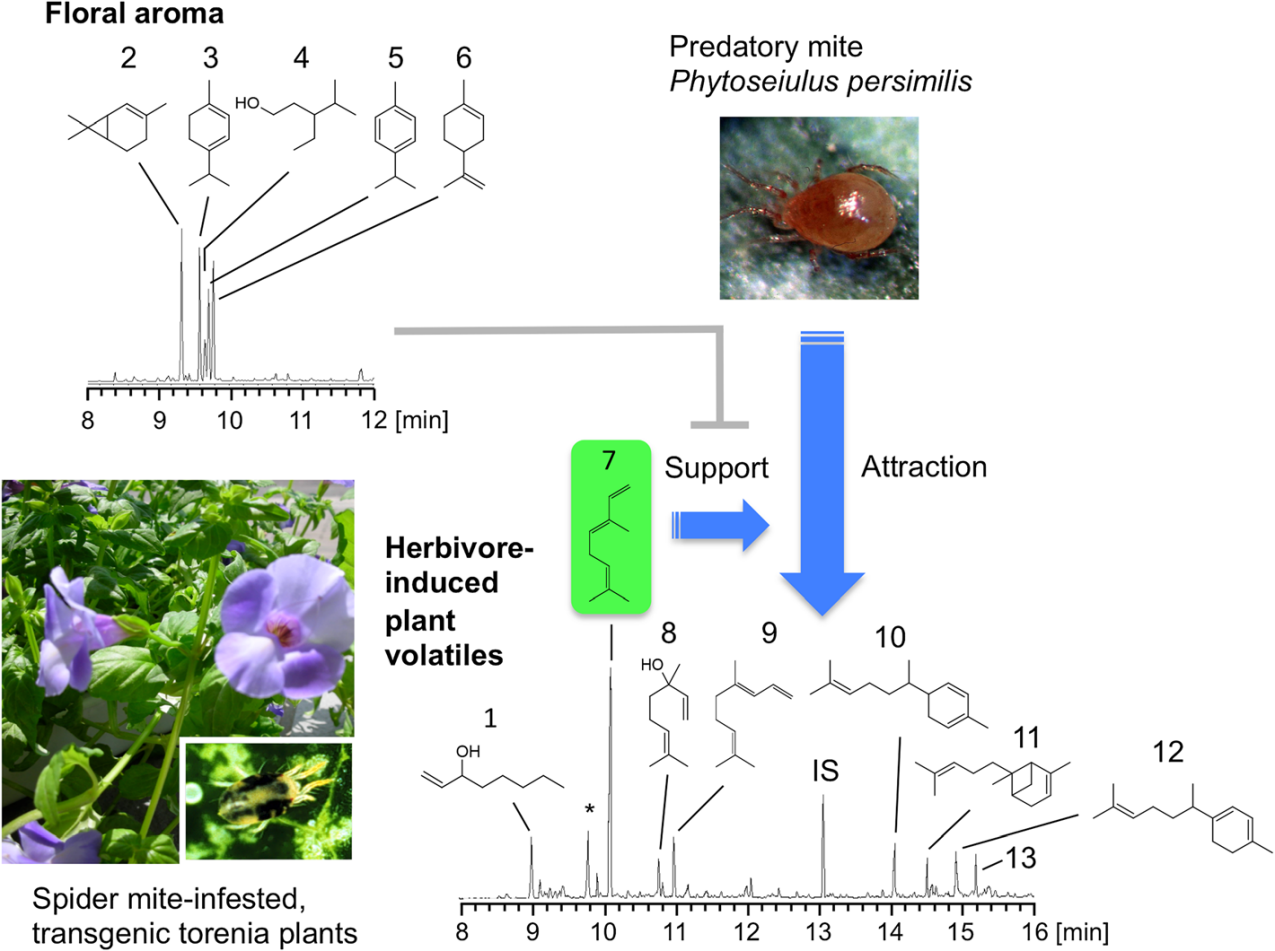
**Tritrophic interaction network in torenia Summerwave blue (*T. hybrida*) for the predatory mite-associated indirect defense responses to spider mites.** Arrows and bars indicate positive and negative interactions, respectively. 1. 1-octen-3-ol; 2. (+)-2-carene; 3. α-terpinene; 4. 3-ethyl-4-methylpentanol; 5. *p*-cymene; 6. limonene; 7. (*E*)-β-ocimene; 8. linalool; 9. (*E*)-4,8-dimethyl-1,3,7-nonatriene; 10. α-zingiberene; 11. α-bergamotene; 12. γ-curcumene; 13. unidentified sesquiterpene; IS, internal standard. An asterisk (*) in volatile profiles indicates air contamination.

Torenia is frequently susceptible to thrips, aphids and mites [1]. Moreover, the accelerated reproductive rate allows these pest populations to adapt quickly to resist pesticides, so chemical control methods can become somewhat ineffectual when the same pesticide is used over a prolonged period. Accordingly, semiochemical-based pest management methods are being developed for ornamental plants with plant volatile-based attractants/repellents that may be used as synthetic chemicals or products of genetic engineering. However, little is known about the characteristics of torenia in ecological interaction networks. To correctly assess the impact of genetically engineered alterations of plant odor in horticultural pest control, it is essential to understand the complicated nature of arthropod attraction mediated by volatile blends. For example, natural enemies of pest herbivores may tend not to respond strongly to novel HIPV blends from transgenic plants at the flowering stage so as to protect themselves against intraguild predation by flower-visiting generalist predators. To evaluate carnivore responses to HIPV blends and to floral volatiles, the ability of transgenic *T. hybrida* plants to attract predatory mites (*Phytoseiulus persimilis*) has recently been studied [15]. The transgenic plants, when infested with two-spotted spider mites (*Tetranychus urticae*), emitted a blend of HIPVs comprising a monoterpene (linalool), a homoterpene [(*E*)-4,8-dimethyl-1,3,7-nonatriene] and sesquiterpenes (α-zingiberene, α-bergamotene, γ-curcumene and unidentified sesquiterpene), as well as a trans-volatile [(*E*)-β-ocimene] (Figure 2) [15]. The trans-volatile enhanced the attraction of *P. persimilis* when added to an HIPV blend from the infested transgenic plants. However, floral volatiles, comprising 3-ethyl-4-methylpentanol and a suite of monoterpenes [(+)-2-carene, α-terpinene, *p*-cymene and limonene], abolished the supporting effect of the trans-volatile embedded in the natural HIPVs. Transgenic torenia thus provides an intriguing tritrophic system, as predator attraction was enhanced only when non-flowering plants were infested (Figure 2) [15]. The attractiveness of a specific volatile compound thus markedly depends on the background odors, including both HIPVs and floral volatiles. Because of such complexity, it is necessary to construct model systems using torenia and other plant species in order to clarify the ecological and horticultural significance of HIPVs.

Metabolic engineering of torenia also demonstrated the abilities of plants to resist fungi and arthropod herbivores. *T. hybrida* manipulated to produce Arabidopsis agmatine coumaroyltransferase (AtACT), which is involved in phytoalexin biosynthesis, was resistant to a necrotrophic fungus, *Botrytis cinerea*, unlike the wild-type progenitors [67]. Collectively, transgenic torenia plants have proven to be an ideal platform for a wide array of pest controls according to the results of various gene manipulations.

## Concluding remarks

In summary, recent advances in torenia research have made this ornamental plant notable for genetic engineering aimed at studying both flower characteristics [5] and pest control [15]. Torenia plants with manipulated flower colors and aroma could be used to investigate interactions between plants and pollinators or floral herbivores. Horticultural ecosystems are, however, very complicated and flexible. For instance, natural enemies of foliage herbivores (folivores) may occasionally be unresponsive to HIPV cues from ornamental plants at flowering stages, so as to protect themselves against intraguild predation by flower-visiting generalist predators. Moreover, damage by folivores can result in delayed and shortened flowering periods and smaller and fewer open flowers. This reduces the floral rewards, such as nectar and pollen, exploited by flower visitors (reviewed in [68]). This is the end result of trade-off between defense and reproduction in plants. Therefore, a wide range of systematic studies using torenia in addition to other plant systems should be conducted to understand realistic horticultural ecosystems, where huge numbers of plants, animals, and microorganisms interact and achieve coevolution and biodiversity.

## Abbreviations

CHS: Chalcone synthase; CRES-T: Chimeric REpressor gene-silencing technology; DFR: Dihydroflavonol 4-reductase; HIPVs: Herbivore-induced plant volatiles; RNAi: RNA interference; VIGS: Virus-induced-gene-silencing.

## Competing interests

The authors declare no competing interests.

## Authors’ contributions

MN and GA conceived the study and drafted the early version of the manuscript. TS and TN commented on the manuscript, revised the text and structure, and outlined it several times together with MN and GA. All authors read and approved the final manuscript.

## Authors’ information

MN’s research focuses on the elucidation of the molecular mechanisms that control flower pigmentation and genetic engineering of novel colored flowers. TS is currently working on behavioral responses of pest herbivores and their natural enemies to herbivore-induced plant volatiles. TN’s research interest is focused on molecular breeding of ornamental flowers. His research interests include plant secondary metabolism, floral morphology and molecular engineering. GA’s research focuses on molecular ecology of plant-insect interactions mediated via volatiles. He has intensively studied mechanisms of resistance to arthropod herbivores.
